# Supplementary material for: WordCommentsAnalyzer: A windows software tool for qualitative research
Source: F1000Res. 2018 Sep 4;7:536. Originally published 2018 May 3. [Version 2] doi: 10.12688/f1000research.14819.2 (PMC6137411; doi:10.12688/f1000research.14819.2)
Supplement: Supplementary file 3 [file f1000research-7-17614-s0002.tgz › b2a79496-4ada-47c4-9682-a99ce867836d.zip!/Eight Iranian Health Journals/Salmand_Iranian Journal of Ageing _19.docx]

© Salmand_Iranian Journal of Ageing . This work is licensed under CC BY-NC 4.0 (https://creativecommons.org/licenses/by-nc/4.0/).

Volume & Issue: دوره 8 - شماره 3

Title: اثربخشی خدمات مراقبتی روزانه مرکز توانبخشی کهریزک کرج بر فعالیت‌‌های روزمره زندگی سالمندان

http://salmandj.uswr.ac.ir/article-1-671-fa.html

اهداف: مراکز توانبخشی تلاش می‌کنند تا با ارائۀ خدمات مراقبتی روزانه به سالمندان و بهبود توانایی‌‌های عملکردی آن‌ها، از ورود غیرضروری به مراکز اقامتی شبانه‌روزی جلوگیری کنند. هدف کلی از این مطالعه، بررسی اثربخشی خدمات مراقبتی روزانۀ مرکز جامع توانبخشی کهریزک کرج بر «فعالیت‌‌های روزمرۀ زندگی» سالمندانی بود که از سال ۱۳۸۹ به‌مدت شش ماه از خدمات گفته‌شده استفاده کرده بودند. مواد و روش‌ها: این مطالعه از نوع پیش‌آزمون‌ و پس‌آزمون بود که براساس اطلاعات موجود در پرونده‌‌های ۷۱ نفر از سالمندان مراجعه‌کننده به مرکز انجام شد. روش نمونه‌گیری تمام‌شماری بود و از شاخص بارتل و مقیاس لاوتون به‌عنوان ابزار‌های مطالعه استفاده شد. سپس، نمرۀ ارزیابی‌‌های «فعالیت‌‌های روزمرۀ زندگی پایه» و «فعالیت‌‌های روزمرۀ زندگی با ابزار» آنان پیش و پس از ارائۀ خدمات مراقبتی روزانۀ مرکز، با یکدیگر مقایسه شد. برای تحلیل داده‌ها از آمار توصیفی، تی‌تست و تحلیل پراکنش و تحلیل هم‌پراکنش استفاده شد. در تمامی موارد، مقدار  (P<۰/۰۰۵) معنا‌دار درنظر گرفته شد. یافته‌ها: میانگین نمره‌های ارزیابی فعالیت‌‌های روزمرۀ زندگی پایۀ سالمندان مطالعه‌شده، پس از دریافت خدمات مراقبتی روزانه با پیش از آن، اختلاف معناداری نشان نداد (۰/۰۹۵=P)؛ درحالی‌که میانگین نمره‌های ارزیابی فعالیت‌‌های روزمرۀ زندگی با ابزارشان، پس از دریافت خدمات مراقبتی روزانه با پیش از آن، اختلاف معناداری نشان داد (۰/۰۰۱>P)‍. از دیگر یافته‌های پژوهش، می‌توان به این‌ها اشاره کرد: بالابودن نمرۀ ارزیابی فعالیت‌‌های روزمرۀ زندگی پایۀ سالمندان موضوع مطالعه‌ که براساس آن، ۷۰درصد از افراد بررسی‌شده دارای حداقل وابستگی بودند و بقیه نیز تنها وابستگی در سطح خفیف داشتند؛ جوان‌تربودن سالمندان مطالعه‌‌شده در مقایسه‌ با جمعیت سالمندی کشور؛ مراجعۀ بیشتر زنان در مقایسه‌ با مردان (سه‌برابر)؛ استفادۀ محدود از وسایل کمک‌توان‌بخشی حرکتی (کمتر از ۱۰درصد) و نبود تفاوت معنادار در نمره‌های ارزیابی فعالیت‌‌های روزمرۀ زندگی پایه در گروه‌‌های سنی گوناگون. نتیجه‌گیری: این مطالعه نشان داد که خدمات مراقبتی روزانه، موجب بهبود فعالیت‌‌های روزمرۀ زندگی سالمندان می‌شود؛ هرچند باتوجه‌ به یافته‌ها، می‌توان گفت ازسویی امکان دسترسی به خدمات روزانۀ در سالمندان با سطح ADL پایین‌تر، کمتر بوده است و ازسوی دیگر، علت اصلی مراجعۀ ایشان، استفاده از خدمات توان‌بخشی روانی، خدمات اجتماعی یا بهبود اوقات‌فراغت بوده است.

Title: موانع و تسهیل‌کننده‌های سالمندان ایرانی در استفاده از دستگاه‌های خودپرداز بانک: یک مطالعه کیفی به‌روش کاوش فرهنگی

http://salmandj.uswr.ac.ir/article-1-688-fa.html

اهداف: باتوجه‌ به سیر افزایشی جمعیت سالمندان در ایران، پیش‌بینی می‌شود طی سی سال آینده، تعداد سالمندانی که نیاز دارند مستقل با وسایل الکترونیکی مانند خودپردازهای بانک کار کنند، رشد چشمگیری بیابد. این در حالی است که هنوز مطالعات کافی به‌منظور مناسب‌سازی این وسایل، باتوجه‌ به توانایی‌های جسمی و ذهنی و روانی سالمندان، انجام نشده است. مواد و روش‌ها: مقالۀ حاضر بخشی از مطالعه‌ای کیفی به‌روش کاوش فرهنگی است که به کشف نیازها و موانع و تسهیل‌کننده‌ها در کار با خودپردازها پرداخته است. داده‌های این مطالعه ازطریق مشاهده و مصاحبه و جمع‌آوری اسناد و مدارک از شرکت‌کنندگان به‌دست آمده است. مطالعۀ کیفی حاضر در دو مرحله انجام شده: ۱. مصاحبه با سی سالمند؛ ۲. مصاحبه با ده سالمند تهران به‌روش نمونه‌گیری هدفمند و استفاده از طراحی بسته‌ای برای استخراج نیازها و مشکلات مربوط‌به خودپردازها. شش نفر از این شرکت‌کنندگان، زن و چهار نفر مرد بودند و مصاحبه‌ها تا مرحلۀ اشباع داده‌ها ادامه یافت. در آخر نیز داده‌ها با روش تحلیل محتوا، کدگزاری و دسته‌بندی و تحلیل شده است. یافته‌ها: شش عامل موردنیاز و اصلی برای طراحی خودپردازها، متناسب با سالمندان ایرانی، از داده‌ها استخراج شد که عبارت است از خوشایندی، ناخوشایندی، خواسته‌ها و آرزوها، مشکلات و موانع، امور بانکی و اتفاقات ناگوار. پس از دسته‌بندی این عوامل، موانع و تسهیل‌کننده‌ها برای سالمندان از آن‌ها استخراج گردید. یافته‌ها نشان داد موانع سالمندان در استفاده از خودپردازها، بسیار بیشتر از تسهیل‌کننده‌ها در وضعیت حاضر است. نتیجه‌گیری: باتوجه‌ به موانع سالمندان ایرانی در استفاده از خودپردازها، پیشنهاد می‌شود دربارۀ این دستگاه‌ها درزمینۀ مناسب‌سازی‌های محیط قرار‌گیری خودپرداز، چگونگی جهت نصب و محل قرار‌گیری، ظاهر خود‌پرداز و به‌خصوص درزمینۀ نرم‌افزارهای آن، مناسب‌سازی‌های لازم متناسب با توان سالمندان انجام شود. درنهایت پیشنهاد می‌شود مطالعات آینده در گروه‌های مختلف سالمندان به‌خصوص سالمندان دچار معلولیت و کم‌سواد انجام شود.

Title: طراحی و روان‌سنجی چک‌لیست شاخص‌های مشاهده‌ای غفلت از سالمندان

http://salmandj.uswr.ac.ir/article-1-583-fa.html

اهداف: پژوهش حاضر با هدف طراحی و تعیین ویژگی‌های روان‌سنجی چک‌لیست شاخص‌های مشاهده‌ای بررسی غفلت از سالمندان انجام شده است. مواد و روش‌ها: مطالعه حاضر پژوهشی روش‌شناختی است که در چهار مرحله انجام شد. در مرحله اول، محققان با استفاده از روش کیفی مفهوم غفلت از سالمندان را تبیین کردند. در مرحله دوم، عبارت‌های چک‌لیست، براساس مصاحبه‌های بدون ساختار با سالمندان و استقرا از گروه متخصصان تدوین شد. سپس در مرحله سوم و چهارم، ویژگی‌های روان‌سنجی پرسش‌نامه با بهره‌گیری از روایی صوری، روایی محتوا، روایی سازه (روایی هم‌گرا و گروه‌های شناخته‌شده)، همسانی درونی و هم‌ارزی بررسی شد. در مرحلۀ اخیر، ۱۱۰ سالمند تهرانی انتخاب شدند و در مطالعه شرکت کردند. یافته‌ها: براساس نتایج حاصل از مرحلۀ اول و دوم مطالعه، چک‌لیست اولیه شامل ۲۶ گویه ارائه شد. پس از تعیین روایی صوری و محتوا به‌صورت کمی و کیفی، گویه‌های چک‌لیست به ۱۱ گویه در دو بُعد غفلت در تأمین نیازهای بهداشتی و مراقبتی و غفلت در تأمین محیط بهداشتی کاهش یافت. ضریب همبستگی پیرسون بین نمره‌های به‌دست‌آمده از چک‌لیست مشاهده‌ای غفلت از سالمندان با مقیاس غفلت مراقبتی پرسش‌نامه بررسی سوءرفتار به سالمندان ۰/۸۶۲ بود. نتایج روش تحلیل مقایسه‌ای گروه‌های شناخته‌شده در پارامترهای وضعیت بستری مجدد، نمایانگر تفاوت بین گروه‌ها بود (۰/۰۰۱>P). محاسبۀ ضریب کودر ریچاردسون (KR۲۰) مؤید همسانی درونی چک‌لیست (۰/۸۲۴) بود. هم‌ارزی ازطریق پایایی بین مشاهده‌کنندگان ۰/۸۵ بود. نتیجه‌گیری: یافته‌ها حاکی‌از آن است که چک‌لیست حاضر با عنوان «چک‌لیست شاخص‌های مشاهده‌ای غفلت از سالمندان» ابزاری مناسب برای بررسی غفلت از سالمندان است.

Title: تأثیر اجرای برنامه آموزش شیوه زندگی سالم بر کیفیت زندگی سالمندان روستایی شهرستان دشتی

http://salmandj.uswr.ac.ir/article-1-615-fa.html

اهداف: امروزه، دغدغۀ اصلی محققان و پژوهشگران صرفاً یافتن راه‌‌های افزایش طول عمر نیست؛ بلکه با نگاهی به آمار و ارقام می‌توان فهمید که اگر در قرن بیستم، چالش اصلی فقط «زنده‌ماندن» بود، چالش قرن جدید، «زندگی با کیفیت برتر» است. هدف این پژوهش، تعیین تأثیر اجرای برنامۀ آموزشی شیوۀ زندگی سالم بر کیفیت زندگی سالمندان روستایی در شهرستان دشتی، در سال ۱۳۸۸ بود. مواد و روش‌ها: طرح این مطالعه از نوع نیمه‌تجربی و پیش‌آزمون‌ و پس‌آزمون و پیگیری همراه با گروه کنترل است. جامعۀ پژوهش حاضر را تمامی سالمندان روستایی شهرستان دشتی تشکیل می‌دهند. نمونه‌‌های پژوهش به‌روش نمونه‌گیری خوشه‌ای چندمرحله‌ای از بین سالمندان ساکن روستا‌های بادوله و تلخو از بخش کاکی شهرستان دشتی استان بوشهر بودند که شرایط ورود به مطالعه را داشتند. افراد نمونه ۷۹ نفر بودند و به‌صورت تصادفی در دو گروه آزمایش و کنترل جای گرفتند. برای جمع‌آوری داده‌ها، از پرسش‌نامۀ سنجش دانش شیوۀ زندگی سالم و پرسش‌نامه 36-SF استفاده شد. داده‌‌های گردآوری‌شده با استفاده از روش‌های آماری تحلیل پراکنش مکرر، تحلیل هم‌پراکنش، آزمون تعقیبی بنفرونی و آزمون کای‌دو تجزیه‌وتحلیل شد. یافته‌ها: نتایج نشان داد آگاهی سالمندان روستایی شهرستان دشتی از شیوۀ زندگی سالم کم بوده و همچنین زندگی آن‌ها بی‌کیفیت است؛ یعنی میانگین نمرۀ کیفیت زندگی قبل از مداخله ۷۶/۶±۸۸/۳۱ است. همچنین، مشخص شد که آموزش شیوۀ زندگی سالم باعث ارتقای میزان آگاهی از شیوۀ زندگی سالم و بهبود کیفیت زندگی سالمندان در گروه مداخله می‌شود (P<0/05). درحالی‌که در گروه کنترل، کیفیت زندگی کاهش می‌یابد؛ گرچه این کاهش به‌لحاظ آماری معنادار نیست و دانش شیوۀ زندگی سالم آن‌ها تغییر معناداری نمی‌کند (P=0/209، f=1/60). نتیجه‌گیری: اجرای برنامۀ آموزشی شیوۀ زندگی سالم باعث بهبود کیفیت زندگی وابسته به سلامت در سالمندان روستایی می‌شود.

Title: مقایسه عوامل خطرآفرین زمین‌خوردن سالمندان با سابقه سقوط ساکن شهر و روستا در شهرستان زابل

http://salmandj.uswr.ac.ir/article-1-571-fa.html

اهداف: هدف اصلی این مطالعه مقایسۀ عوامل خطرآفرین زمین‌خوردن در سالمندان با سابقۀ سقوط ساکن شهر و روستای شهرستان زابل بود. مواد و روش‌ها: مطالعۀ حاضر بررسی توصیفی‌تحلیلی مقطعی بر نمونه‌ای متشکل از ۱۷۳ سالمند بدون اختلال شناخت، ساکن در شهر و روستاهای شهرستان زابل و با سن بیش از ۶۰ سال در سال ۱۳۸۹تا۱۳۹۰ است. ابزار گرد‌آوری داده‌ها پرسش‌نامه بود. اطلاعات به‌دست‌آمده با استفاده از نسخۀ ۱۶ نرم‌افزار آماری SPSS تحلیل شد. برای تجزیه‌وتحلیل داده‌های بخش دموگرافیک، از شاخص‌های مرکزی و پراکندگی آمار توصیفی و برای مقایسۀ میانگین عوامل خطرآفرین و نحوۀ زمین‌خوردن و تکرار آن برحسب متغیرها، از آزمون‌های تی و تحلیل پراکنش و کای‌دو در سطح آماری معلوم (0/5>P) استفاده شد. یافته‌ها: از میان عوامل داخلی، دیابت (0/001=P)، مشکل ریوی (0/011=P)، مشکل شنوایی (0/017=P)، مشکل قلبی (0/018=P)، سابقۀ جراحی (0/025=P) و فشارخون بالا (0/03=P) به‌طور معناداری در سالمندان شهری بیشتر از سالمندان روستایی بوده است. از میان عوامل خارجی، میزان فعالیت ورزشی به‌صورت معناداری در سالمندان شهری بیشتر از روستایی بود (0/5>P) و تفاوت معناداری در نحوۀ سقوط سالمندان شهری و روستایی مشاهده نشد (0/239=P) و وضعیت سالمندان بعد از سقوط، در شهر و روستا مشابه بود. نتیجه‌گیری: نتایج این پژوهش نشان می‌دهد انواع بیماری‌های دیابت، مشکلات ریوی، مشکلات شنوایی، مشکلات قلبی و سابقۀ جراحی، از دلایل عمدۀ زمین‌خوردن سالمندان شهری بوده است. میزان فعالیت ورزشی در سالمندان شهری بیش از روستایی بود و مکان عمدۀ سقوط، در مکان‌های حیاط‌دار حیاط بود و پس از آن، داخل اتاق. همچنین، زمان عمدۀ سقوط سالمندان، صبح بوده است. بنابراین، افزایش فعالیت‌های بدنی برای افزایش سلامت سالمندان شهری و کاهش سقوط آنان کافی نبوده و کاهش آمار سقوط، نیازمند توجه و مراقبت در ابعاد وسیع‌تری ازجمله کنترل بیماری‌های همراه و توجه به مکان زندگی سالمند و رفع عوامل خطر محیطی است.

Title: تأثیر توانبخشی حافظه و توجه در کاهش نقایص حافظه سالمندان مبتلا‌ به دمانس آلزایمر

http://salmandj.uswr.ac.ir/article-1-604-fa.html

اهداف: دمانس ناشی از بیماری آلزایمر، یکی از مشکلات شناختی بسیار مزمن‌ در بین سالمندان است که تأثیرات روانی و اقتصادی فراوانی بر دوش بیمار و خانوادۀ او می‌گذارد. در این پژوهش، به بررسی تأثیر توان‌بخشی حافظه و توجه در کاهش نقایص حافظۀ بیماران پرداخته‌ایم که این روش، رویکردی غیرزیستی و نوین در درمان بیماری آلزایمر است. مواد و روش‌ها: این پژوهش جزو مطالعات شبه‌تجربی از نوع طرح‌های تک‌آزمودنی و سری‌های زمانی A-B با گروه کنترل و دستۀ درون‌سری‌ها بود. ۸ بیمار مبتلا‌به آلزایمر خفیف تا متوسط، با روش نمونه‌گیری در دسترس انتخاب شده و به دو گروه آزمایش و گواه، تقسیم شدند. مدت پژوهش ۱۴جلسه بود. برای گروه آزمایش، ۱۲ جلسۀ توان‌بخشی (هفته‌ای ۲ جلسه) به‌مدت ۴۵ دقیقه تا ۱ ساعت، اجرا شد. برای همتاسازی آزمودنی‌ها، از آزمون‌های MMSE و 15-GDS استفاده شد. برای بررسی نتایج، آزمودنی‌ها هر ۴ جلسه (در جلسات ۱، ۴، ۸ و ۱۲) توسط خرده‌مقیاس‌های حافظۀ منطقی و یادگیری تداعی‌های WMS-O ارزیابی شدند. تجزیه‌وتحلیل داده‌های پژوهش، هم در سطح بین فردی (زوج‌های همتا) و هم به‌صورت گروهی، با استفاده از تحلیل چشمی و محاسبۀ درصد بهبود و اندازۀ اثر صورت گرفت. یافته‌ها: نمودارهای ترسیم‌شده و تحلیل‌های آماری نشان‌دهندۀ آن بود که آزمودنی‌های گروه آزمایش، چه درمقایسه‌ با آزمودنی همتا و چه در مقایسۀ گروهی، نسبت‌به خط پایه تغییر و بهبود چشمگیری در نمرات نشان دادند. اندازۀ اثر محاسبه‌شده برای کارکرد حافظۀ گروه آزمایش (3/11-3/17=d)، بسیار بیشتر از اندازۀ اثر گروه گواه (۰/۶۲-۰/۸۸) بود. همچنین، سطح تغییرات وضعیت حافظۀ آزمودنی‌های گروه آزمایش از خط پایه، معنادارتر از گروه گواه بود. نتیجه‌گیری: می‌توان چنین گفت که ترکیب توان‌بخشی حافظه و توجه درمقایسه‌ با بهره‌گیری از توان‌بخشی با هریک به‌تنهایی، نتایج مطلوب‌تری داشته و در زمان کوتاه‌تری، موجب کاهش نقایص حافظۀ سالمندان مبتلا‌به آلزایمر شده است. بنابراین، با ترکیب توان‌بخشی این دو حوزۀ مهم شناختی، دامنۀ گسترده‌ای از مؤلفه‌های شناختی بیماران، تحت بازتوانی قرار می‌گیرد و به نتایج مطلوب‌تری می‌توان دست یافت.

Title: روایی و پایایی مقیاس جهانی ارزیابی دمانس رولاند در نمونه‌ای از زنان سالمند

http://salmandj.uswr.ac.ir/article-1-535-fa.html

اهداف: هدف از مطالعۀ حاضر، بررسی اعتبار و پایایی مقیاس جهانی ارزیابی دمانس رولاند است که در نمونه‌ای از زنان سالمند بررسی شده است. مواد و روش‌ها: این مطالعه پیرو طرحی از نوع توصیفی بوده و پس از اخذ رضایت شرکت در تحقیق، روی ۳۱۰ زن سالمند از دو گسترۀ آسایشگاه کهریزک تهران و کانون جهان‌دیدگان تهران انجام شده است. در این پژوهش، نمونه‌ها به‌صورت نمونه در دسترس، انتخاب و برای جمع‌آوری اطلاعات، از چهار پرسش‌نامه استفاده شد که به‌صورت مصاحبه با سالمند تکمیل شده است. اطلاعات ازطریق نرم‌افزار ۱۶spss و با استفاده از روش‌های آماری مربوط، تجزیه‌وتحلیل شد. یافته‌ها: براساس یافته‌های تحقیق، میانگین سِنی افراد موضوع مطالعه، ۷۶/۸۶ سال بوده است. اعتبار مقیاس آزمون به روش هم‌گرا با پرسش‌نامه‌های کیفیت زندگی و افسردگی سالمندان و روش واگرا با پرسش‌نامه‌های محور افت شناختی سالمندان و معاینۀ مختصر وضعیت شناختی بررسی شد. همچنین، پایایی مقیاس به روش آلفای کرونباخ، ۰/۷۹۲ و روش دونیمه‌کردن، ۰/۷۵۹ و آزمون‌بازآزمون، ۰/۷۶ بوده است. همچنین، تحلیل عاملی بیانگر اشباع این مقیاس از یک عامل بوده است. نتیجه‌گیری: نتایج مطالعه یادشده، بیانگر این مطلب است که این مقیاس در جمعیت زنان سالمند، اعتبار و پایایی مطلوبی دارد. به‌نظر می‌رسد برنامه‌ریزی برای اجرای این پژوهش، در جمعیت‌های بالینی و مختلط بسیار مؤثر خواهد بود.

Title: مقایسه کیفیت زندگی افراد سالمند ورزشکار و غیرورزشکار شهر تبریز

http://salmandj.uswr.ac.ir/article-1-453-fa.html

اهداف: این تحقیق با هدف بررسی کیفیت زندگی افراد سالمند ورزش‌کار و غیرورزش‌کار و مقایسۀ بین آن‌ها انجام شد. مواد و روش‌ها: تحقیق حاضر ازنظر هدف، کاربردی و از‌نظر ماهیت و روش، توصیفی است. ۱۸۹ نفر از افراد سالمند شهر تبریز به‌عنوان نمونۀ آماری انتخاب شدند. برای نمونه‌گیری از روش تصادفی استفاده شد و ابزار جمع‌آوری داده، پرسش‌نامۀ مشخصات فردی و پرسش‌نامۀ کیفیت زندگی براساس الگوی واروشربون بود. همچنین، از روش‌های آماری‌توصیفی و کلموگروف‌اسمیرنف و مان ویتنی و تی تست استفاده شده است. یافته‌ها: در مؤلفه‌های کیفیت زندگی سالمندان ورزشکار و غیرورزشکار در سلامت عمومی، عملکرد جسمانی، انرژی و شادابی، سلامت روانی و محدودیت‌های ایفای نقش به‌دلایل جسمانی و عاطفی اختلاف معنی‌دار وجود دارد؛ اما در متغیرهای درد بدنی و عملکرد اجتماعی اختلاف معنی‌داری مشاهده نشد. نتیجه‌گیری: تحلیل نتایج، نشان‌دهندۀ تأثیر فعالیت بدنی در بیشتر متغیرهای بررسی‌شده در سالمندان است. اجزای تشکیل‌دهنده بهبود توانایی عملکرد به‌صورت مجزا و ترکیبی کمک می‌کند سالمندان بتوانند دامنه‌ای از حرکات روزمرۀ مورد‌نیاز را انجام دهند و کیفیت زندگی خود را در ابعاد جسمانی و روانی افزایش دهند. در‌خصوص عملکرد و تعامل اجتماعی سالمندان، باید برنامه‌ریزی منسجم و مؤثر شود. ایجاد انگیزۀ ورزش برای آنان نیز باید با برنامه‌ریزی و تأکید بر تنوع در فعالیت‌ها و تقویت نقاط مثبت و استفاده از طرح‌های خلاقانه و ابداعی همراه باشد.
